# Influence of the recent winter Arctic sea ice loss in short-term simulations of a regional atmospheric model

**DOI:** 10.1038/s41598-022-12783-4

**Published:** 2022-05-26

**Authors:** Heeje Cho, Jong-Seong Kug, Sang-Yoon Jun

**Affiliations:** 1 Division of Atmospheric Sciences, Korea Polar Research Institute, Incheon, 21990 South Korea; 2 Division of Environmental Science and Engineering, Pohang University of Science and Technology, Pohang, 37673 South Korea

**Keywords:** Atmospheric dynamics, Climate and Earth system modelling

## Abstract

Notable changes in the wintertime Arctic atmospheric circulation have occurred over the last few decades. Despite its importance in understanding the recent changes in the Northern Hemisphere midlatitude climate, it remains unclear whether and how these changes are affected by recent Arctic sea ice loss. In this study, a regional scale model is used to separate the direct sea ice influence from the natural variability of large-scale atmospheric circulation. Results show that, in response to sea ice loss, the increase of geopotential height in the mid-to-upper troposphere is robust across the simulations, but the magnitude of the response is highly dependent on the background state of the atmosphere. In most cases the sea ice loss-induced atmospheric warming is trapped near the surface due to the high vertical stability of winter Arctic lower troposphere, accordingly, resulting in a small response of geopotential height. However, when a low-pressure system is located over the Barents Sea, the relatively weak stability allows an upward transport of the surface warming, causing a significantly larger geopotential height increase. This strong state-dependence of atmospheric response which is also found in recent studies using global-scale model experiments, highlights the importance of accurately representing the atmospheric background state for numerical model assessments of sea ice influence.

## Introduction

Observations have revealed an ongoing rapid surface warming and sea ice loss over the Arctic region^1–3^. For the winter season, the warming trend was relatively weak until the late 1990s, but it has become rapidly accelerated since then^1–4^. Meanwhile, the winter Arctic atmosphere has shown a notable increase in atmospheric pressure and associated anticyclonic circulation anomalies^5^. The change is particularly large and statistically significant over the Barents Sea region where winter sea ice has exhibited the strongest declining trend^6^. However, it is unclear whether the atmospheric circulation changes over the Barents Sea are a response to the underlying sea ice change or not^6–8^.

This is difficult to address because there exists positive feedback between the two processes: Arctic atmospheric circulation changes and Arctic sea ice loss. That is, these two processes can be induced by each other. For instance, excessive surface heat release due to sea ice loss results in a local increase of atmospheric pressure^9,10^, and also, an anomalous high-pressure near the Barents–Kara Sea is known to accelerate sea ice melting by increased horizontal heat and moisture transports into the Atlantic sector of the Arctic^11–13^. Because of the two-way interaction, the role of sea ice loss in the atmospheric circulation changes is difficult to identify in observational data. Moreover, not only Arctic sea ice, various factors that could affect the Arctic atmospheric circulation, such as ocean circulation and global-scale atmospheric circulation, also have experienced significant changes during the recent decades^14–16^.

This is an important issue in understanding the recent cooling trends of land surface temperature and the increased occurrence of extreme cold events in the Northern Hemisphere winter. It has been suggested that the Arctic warming from sea ice loss can elicit a large scale atmospheric circulation anomaly (e.g., strengthening and/or expansion of Siberian High) that cools the midlatitude surface, forming the so-called “warm Arctic-cold continents pattern”^17^, but whether it is caused by the Arctic sea ice reduction is still a subject of debate^18–21^. Recent studies have argued the statistical significance of the increasing winter extreme events, questioning the role of sea ice loss and examining the effects of internal variability and the amplified Arctic surface response to anthropogenic global warming^22–24^.

Numerical models can be used to extract the contribution of recent sea ice changes by comparing two identical simulations but with different sea ice states. However, it has been pointed out that the uncertainty in Arctic climate modeling is high with Earth system models having different representations of dynamical and physical processes^25,26^. Recent studies under the Polar Amplification Model Intercomparison Project (PAMIP) protocol^27^ have also reported that uncertainties could be attributed to other factors such as internal variability of the stratospheric polar vortex^28^, the ice-constraining method^29^, and an insufficient number of ensembles^30^. Also, note that the sea ice loss impact can be sensitive to experimental design because the additional turbulent heat flux from sea ice loss is known to vary with changing atmospheric conditions^31^. In Earth system model simulations, responses to the Arctic sea ice forcing can also develop outside the Arctic region. The tropics and midlatitudes responses eventually interact with the Arctic atmosphere, hence the interaction may blur signals of the direct response of the Arctic atmosphere to sea ice forcing. This is because the interaction with lower latitudes is dependent on the mean climate state which differs greatly from model to model. Consequently, in Earth system models, various factors can contaminate sea ice-forced signals, leading to large inter-model differences.

As an alternative, a regional-scale atmospheric model (Polar WRF)^32,33^ is used in this study to assess the direct influence of the wintertime Arctic sea ice loss on the Arctic atmospheric circulation. The main advantage of employing a regional model is that we can use a short model integration time (48 h in this study) so that the sea ice-forced signals in the atmosphere can be distinct before they are mixed with the interaction with large-scale atmospheric disturbances. This is difficult for a global-scale model simulation which requires a longer initial spin-up. Note that in some studies global-scale models have been utilized to examine the sea ice influence on a relative short-term scale (< 1 month)^31,34,35^, however, regional-scale models have rarely been used to conduct a sensitivity experiment with modified sea ice fields. Previous Polar WRF studies whose simulation setup is similar to that of this study have demonstrated that the model can successfully reproduce the winter Arctic climate^32,36,37^. Also, unlike global-scale models, regional simulations require lateral boundary conditions which are updated every 6 h in this study providing additional constraints to simulations. Therefore, interactions with lower latitudes can be controlled by observational information. Furthermore, the influence of initial conditions helps keep the simulated climatology more realistic which will prove to be important in this article.

## Results

In the current study, we conducted two sets of simulations in which the simulation setups are identical to each other except for the mean sea ice concentration (SIC). The SIC difference at each grid point is based on observed mean SIC differences between two periods, 1979/80–1988/89 and 2006/07–2015/16. From the difference between the two sets of simulations, we expect that changes that are attributed only to the climatologically reduced sea ice can be obtained. Each simulation set consists of 903 realizations; each realization is a 48-h simulation initialized at 00 UTC every day during 10 winter seasons (December–February from 2006/2007 to 2015/2016). Note that the two simulation sets use the same sea ice albedo and thickness data which are temporally and spatially varying estimates from satellite observations. By generating the large ensemble of realizations this way, the simulated ensemble average can be representative of the winter Arctic climate, and the signal-to-noise ratio of atmospheric response to sea ice loss can be increased which is known to be low due to large internal variability.

**Figure 1:**
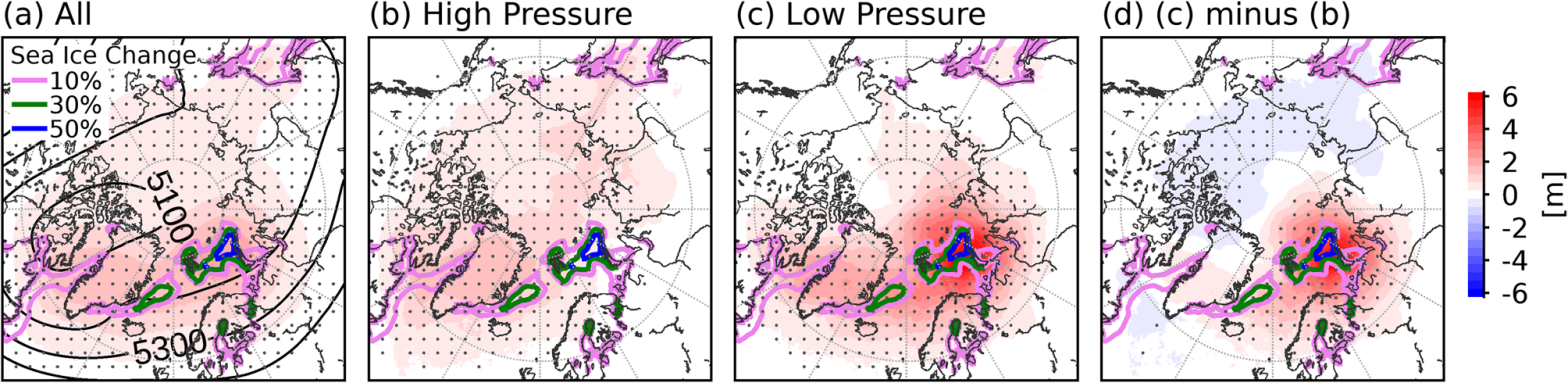
(**a**–**c**) Responses of wintertime (Dec–Feb) 500 hPa geopotential height to sea ice loss for (**a**) all “control” run period (2006/07–2015/16), (**b**) “high-pressure” cases, and (**c**) “low-pressure” cases. The response is obtained by subtracting the “high sea ice concentration (High SIC)” run from the “control” run of Polar WRF. The “control” run is an ensemble of 48-h simulations initialized daily during the period and the “High SIC” run is identical to the “control” run but with adding the wintertime sea ice concentration difference between two periods (1979/80–1988/89 minus 2006/07–2015/16). “High-pressure” and “Low-pressure” cases are days with the top and bottom 5% of the 500 hPa geopotential height over the Barents Sea region (70–80°N, 15–75°E). (**d**) Difference between the mean response of the “high-pressure” cases and the mean response of the “low-pressure” cases. The values are daily averages at day 2 of model integration (24-to-48-h). Differences that are statistically significant based on a Student’s *t* test at the 99% confidence level are stippled. Colored contours indicate the SIC differences between the “control” and “High SIC” runs. Black contours in (**a**) are the mean 500 hPa geopotential field for the control run. The map has been created using Matplotlib Basemap Toolkit ver. 1.3.0 (https://matplotlib.org/basemap/).

Figure 1a shows the mean difference of 500 hPa geopotential height (∆z500) at day 2 of model integration (24-to-48-h), which can be regarded as an “immediate” responses to the sea ice changes. There are positive geopotential height anomalies over most of the Arctic, and they are statistically significant at the 99% confidence level (stippled regions in Fig. 1a), indicating sea ice loss plays a role in increasing atmospheric pressure of the middle troposphere. In particular, the response is strongest around the Barents Sea (about 2–3 m), where sea ice loss has been strongest (colored lines in Fig. 1a). Note that additional heat transferred to the atmosphere is larger over the Barents Sea because of its climatologically warmer sea surface temperature than other regions in the Arctic. The strong responses are also found over the western and eastern sides of Greenland (the Baffin Bay, the Labrador Sea, and the Greenland Sea) and over the Sea of Okhotsk where sea ice loss has been significant. The similarity between the spatial patterns of the sea ice forcing and ∆z500 indicates an immediate atmospheric response to sea ice forcing. The positive geopotential response over the Barents Sea has consistently appeared in most 903 realizations of 48-h simulation with few exceptions, suggesting the robustness of the response. Though the sign of ∆z500 is positive with very high confidence, the magnitudes of ∆z500 are quite diverse with a standard deviation of about 1.6 m near the Barents Sea. The spread in ∆z500 comes mostly from synoptical variability of the atmosphere. Note that the standard deviation of the seasonal mean ∆z500 is about 0.5 m near the Barents Sea, and ∆z500 fields in each month during winter (Fig. S1) are similar to Fig. 1a, confirming the representativeness of the response in Fig. 1a.

Our analysis reveals that the magnitude of the atmospheric response is well explained by the geopotential height of the mid- to upper-troposphere. Accordingly, based on the simulated 500 hPa geopotential height over the Barents Sea region (70–80°N, 15–75°E), high-pressure and low-pressure cases are defined as the highest and lowest 5% of all the 903 realizations. For the high-pressure cases (Fig. 1b), ∆z500 is generally small over the Barents Sea. While the responses are statistically significant, their magnitudes are not particularly large but similar to the average Arctic responses. On the other hand, the low-pressure cases show distinctly larger values of ∆z500 (Fig. 1c). Over the Barents Sea region, the geopotential height increase of the low-pressure cases is about 6 m which is 12 times larger than that of the high-pressure cases; this is about 10% of the interannual variation of the winter mean 500 hPa geopotential height over the region. The difference between the mean high-pressure response and the mean low-pressure response is also statistically significant over the Barents Sea region (Fig. 1d). This noticeable difference between the high- and low-pressure cases suggests that the effect of sea ice loss on the atmosphere is highly sensitive to background atmospheric conditions.

**Figure 2:**
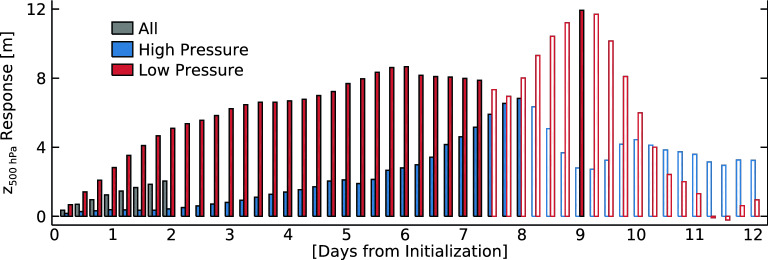
Evolution of the 500 hPa geopotential response over the Barents Sea region (70–80°N, 15–75°E) during the model integration time for all (gray), “high-pressure” (blue), and “low-pressure” (red) cases. Filled (blank) bars represent responses that are (not) significant based on a Student’s *t* test at the 99% confidence level.

Figure 2 shows the temporal evolution of the atmospheric response during model integration. The values are ∆z500 averages over the Barents Sea region of 70–80°N, 15–75°E. In general, the response is gradually amplified with the accumulation of the sea ice effects until about 6 to 8 days of model integration. Notably, the responses are much larger for the low-pressure cases (red bars in Fig. 2) than the average responses (gray bars in Fig. 2), reaching 8 m at day 6 of model integration. In comparison, the high-pressure cases (blue bars in Fig. 2) show order-of-magnitude smaller responses than the low-pressure cases at day 2 of model integration. Although high-pressure case responses show gradual increases with time, this is likely because anomalous high pressure in the mid- to upper-troposphere weakens with time due to the natural synoptic-scale variations. This is also the reason why the responses beyond 7 days become unstable and statistically insignificant for both high- and low-pressure cases. An important implication of the insignificant responses in a long model integration time is that the direct influence of sea ice loss is not strong enough to sustain a detectable atmospheric response in the presence of the natural variability of the atmospheric circulation. However, it should be noted that, at day 7 of model integration, the atmospheric response is statistically significant over most of the Arctic Ocean for the low-pressure cases, while it is only near the East Siberian Sea for the high-pressure cases (Fig. S2).

**Figure 3:**
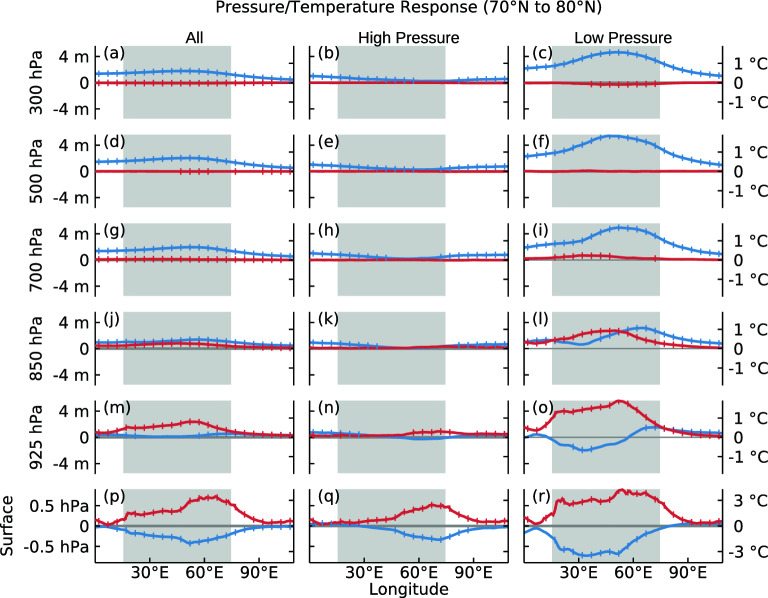
Zonal structure near the Barents Sea region (averages for 70–80°N) for all (**a**, **d**, **g**, **j**, **m**, **p**), “high-pressure” (**b**, **e**, **h**, **k**, **n**, **q**), and “low-pressure” (**c**, **f**, **i**, **l**, **o**, **r**) cases. Red lines indicate temperature response. Blue lines indicate surface pressure responses at surface level (**p**, **q**, **r**) and geopotential height responses in the atmosphere (**a**–**o**). The left y-axes are surface pressure in hPa (**p**, **q**, **r**) and geopotential height in meters (**a**–**o**). The right y-axes are temperature, and note that the scale for surface (**p**, **q**, **r**) is different from that for atmopshere (**a**–**o**).

Figure 3 displays the vertical structure of atmospheric responses to sea ice loss over the Barents Sea region. The values are meridional averages between 70°N and 80°N. The surface temperature increase, maximized at 50–60°E (red lines in Fig. 3p), is a direct response to the additional heat fluxes from sea ice loss. The resultant atmospheric warming signals are found in the lower troposphere below 700 hPa (red lines in Fig. 3j,m). It is interesting that surface pressure decreases in response to surface heating (blue line in Fig. 3p). The decreased surface pressure and decreased geopotential heights near the heating level are also present in an idealized simulation study by Lin^38^ which examined responses of a two-dimensional atmosphere with a uniform flow when concentrated steady heating is applied. The geopotential height response becomes near zero at 925 hPa (blue line in Fig. 3m), and in the upper atmosphere, it changes to positive and intensifies with height (blue lines in Fig. 3a,d,g,j). The spatial fields of the vertical structure of the responses are shown in Fig. S3.

The atmospheric responses can be understood as an atmosphere's hydrostatic adjustment to a surface or planetary boundary layer (PBL) heating. The positive buoyancy at the low-level troposphere produced by surface heating can elevate the geopotential heights of the atmosphere above the heating level. Accordingly, near and below that level, negative geopotential height anomalies are induced to compensate for the upper-level changes. This “anticyclonic above-cyclonic below” response has been rarely found in monthly or seasonally averaged data. This is because while the initial immediate atmospheric responses have a baroclinic structure, the synoptic eddy feedback tends to induce a barotropic anomaly structure by a potential eddy-vorticity flux^39^ so that lower atmospheric circulation anomaly eventually has the same sign with upper-level. In other words, the present study succeeded in capturing the direct responses of the atmosphere to surface heating because we used short-time weather forecast-scale simulations.

The dependency on the atmospheric background state is also shown in Fig. 3. The high-pressure cases (second column of Fig. 3) show very small responses throughout the atmosphere even in the sea ice loss region around 50–60°E, but the temperature increase and pressure decrease are visible only at the surface level (Fig. 3q). In contrast, responses for the low-pressure cases are notably larger (third column of Fig. 3). The strong warming at the surface extends up to the 850 hPa level. The surface and atmospheric responses are also intense, showing large decreases in surface pressure and 925 hPa geopotential height and large increases in the upper-level geopotential heights.

Another important feature that should be noted in Fig. 3 is the westward tilt of geopotential responses. This is clearly visible for the low-pressure cases (third column of Fig. 3). Negative surface pressure and 925 hPa geopotential responses are found near or west of the surface heating region (Fig. 3o,r), whereas at 850 hPa, a positive geopotential response is produced east of the surface heating region, and it moves westward with height. Also, note that the geopotential response shows a noticeable amplification with height. The upstream phase tilt of the responses is also a characteristic feature found in the previous idealized model study^38^, along with the decreased pressure response at the heating level (Fig. 3p–r) and the increased geopotential height response downstream of the heating region (Fig. 3l,o). While the tilting can be explained as a vertically propagating gravity wave^38^, another interpretation is also possible. When a steady surface boundary heat forcing is applied, the atmospheric heating due to vertical heat transport can be balanced by a horizontal advection in order for the atmospheric response to be steady. In such a case, cold advection by northerly wind may be the most efficient way to balance the vertical heat transport, which favors a westward tilt with height.

**Figure 4:**
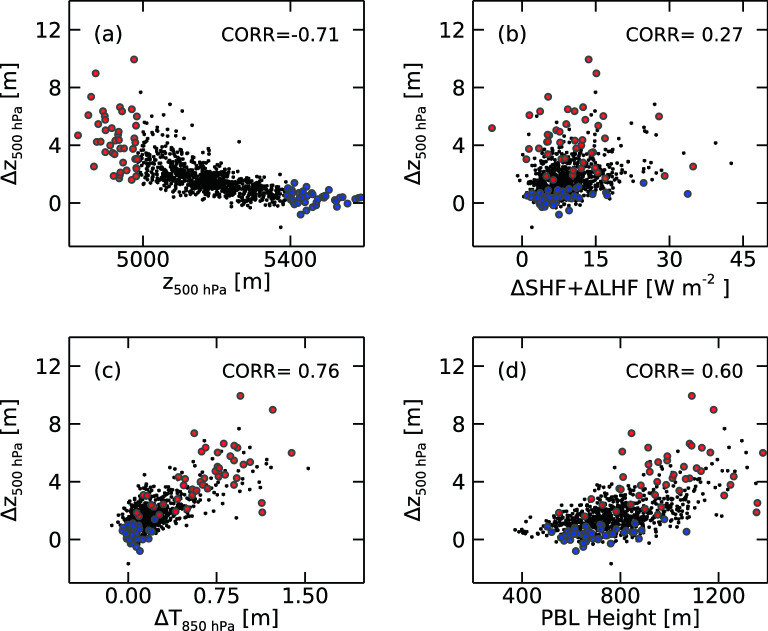
Dependency of the 500 hPa geopotential height response on (**a**) 500 hPa geopotential height, (**b**) responses of the sensible and latent heat fluxes at the surface, (**c**) 850 hPa air temperature response, and (**d**) planetary boundary layer height over the Barents Sea region (70–80°N, 15–75°E). All 903 realizations are plotted. Red and blue circles represent the low- and high-pressure cases, respectively.

As implied by the significantly different responses between high- and low-pressure cases, the local atmospheric pressure is important in controlling the strength of the atmospheric responses. This is shown in Fig. 4a which displays a strong linear correlation between ∆z500 (atmospheric response) and 500 hPa geopotential height (local atmospheric pressure) over the Barents Sea region. With regard to forcing, sea ice changes can affect the atmosphere most effectively by regulating surface turbulent processes in the form of sensible or latent heat fluxes (Fig. S4). However, in our simulations, the response of the total (sensible + latent) turbulent heat flux is positively but weakly correlated with ∆z500 (r = 0.27) (Fig. 4b). Note that the additional surface turbulent heat for the low-pressure cases (10.6 W m^−2^) is only larger by 36% than that for the high-pressure cases (7.8 W m^−2^), which cannot fully account for the stronger response of low-pressure systems. Interestingly, ∆z500 is remarkably well explained by low-level atmospheric heatings, showing r = 0.76 with 850 hPa atmospheric temperature responses (Fig. 4c). By comparison, the surface temperature response showed a smaller correlation coefficient of 0.44 with ∆z500. This may be because the atmospheric response is more sensitive to the heating at a height where the wind is strong enough for vertical wave propagation. In this regard, low-level stability can be important in controlling the upward transport of surface heat, particularly in the wintertime Arctic where the upward heat transport can be severely suppressed by the stable low-level atmosphere due to the radiative cooling-induced cold surface temperature.

PBL height involves the model’s bottom-level stability and also the turbulent activity near the sea surface. Note that the daily mean PBL height of the Barents Sea rarely exceeds 1.2 km during the entire 10 winter seasons (Fig. 4d). The mean responses in PBL height are large in sea ice loss regions (Fig. S5) and their spatial patterns are similar to those of the responses in surface turbulent heat fluxes (Fig. S4). The result revealed that ∆z500 is well explained by PBL height showing a strong linear correlation (r = 0.60, Fig. 4d). This is because PBL height tends to be larger for a low-pressure system resulting in stronger turbulent activity and/or a more unstable near-surface atmosphere which helps transport the surface heat up to a higher level. It is important to note that the PBL height is well correlated with the mid- to upper-level geopotential height showing a significant negative correlation of − 0.48 with 500 hPa geopotential height, while, on the other hand, it is not with surface temperature (r =  − 0.04) or with sea-level pressure (r =  − 0.19). This implies that the upper-level synoptic condition of the Arctic region is important in controlling the sensitivity of the atmospheric response to the sea ice change, justifying our use of 500 hPa geopotential heights for classifying local meteorological conditions of the Barents Sea region.

## Discussion

In summary, the direct influence of winter Arctic sea ice loss on the atmosphere is robust but small in magnitude because of suppressed upward heat transport by the very stable winter Arctic lower troposphere. However, when a low-pressure system is located over sea ice loss regions, the reduced stability allows a considerably larger response in the atmosphere. This is demonstrated in the present study by utilizing a large set of short-time simulations of a regional model, which might be difficult with longer time scale simulation of global-scale models. The atmospheric responses in our results are not large enough to account for the recent Arctic circulation changes, given that 500 hPa geopotential height over the Barents Sea region has increased by about 60 m for the last three decades. This is partly because the 48-h model integration time is too short to produce a dynamical interaction with large-scale circulation fields. Also, the lateral boundary conditions inhibit interactions with synoptic-scale eddy fluxes from lower latitudes. Moreover, in reality, the low-pressure anomaly over the Barents Sea is known to increase the influx of sensible heat and moisture from lower latitudes that further melt the underlying sea ice. This positive feedback process between sea ice and circulation anomaly is not allowed due to the prescribed surface boundary conditions. While the resulting small but robust geopotential height response is an essential prerequisite for producing remote responses in midlatitude regions, it is beyond the scope of the present study to examine whether the atmospheric responses can grow in strength, excite remote responses in midlatitude, and have seasonal and longer timescale influences in the Northern Hemisphere midlatitudes. Nonetheless, the short-term regional simulation benefits from the initial and lateral boundary conditions so the simulations can be closer to the observed Arctic winter climate than those from long-term global simulations. This turns out to be crucial in obtaining a reliable response considering our result showing that responses of the atmosphere are highly sensitive to local meteorological conditions such as mid- to high-level geopotential height and low-level static stability. Note that the fidelity of global-scale models in representing the mean climate state and its variability has been an important issue for understanding the sea ice influences on the global climate^40,41^.

We also found that the atmospheric warming due to sea ice loss appears only near the surface level in most cases, but it reaches 700 hPa level for the low-pressure cases. In other words, the vertical extension of the warming response to sea ice loss is also sensitive to the background state. This has implications for the recent Northern Hemispheric cooling trend because, as pointed out by a recent study^42,43^, the vertical extent of warming over the Barents Sea is important in understanding the Arctic-midlatitude linkages in Earth system model simulations. While it is beyond the scope of the present paper, further research on why only for the low-pressure cases, the Arctic-wide responses can develop in time (Fig. S2) would help understand the issue. Our results suggest that the large discrepancies in the midlatitude responses to sea ice loss across Earth system models are caused by model-to-model variations in background states which are typically larger in high-latitude regions in winter^44^, highlighting the importance of accurate simulation of Arctic mean climate, especially near the surface, for obtaining reliable midlatitude responses.

It is shown here that the geopotential height increases are an order of magnitude larger for the low-pressure cases than for the high-pressure cases. This means that Arctic sea ice loss can be effective at weakening low-pressure systems but has little influence on high-pressure systems. Therefore, the sea ice influence can be more evident in some cases than others, and it can be underestimated when monthly or seasonally averaged fields of observational or simulated data are used. In this context, it is possible that Arctic atmospheric geopotential height responses to sea ice loss are stronger for a certain phase of the wintertime Arctic Oscillation or North Atlantic Oscillation. This state-dependence found in the short-term simulations may cause the different hemispheric influence of the sea ice loss for different climate state, which has been revealed in a large ensemble experiment with a global-scale climate model^45^. Furthermore, as the Arctic surface warming continues in the future, the near-surface atmosphere would be more unstable, consequently, the Arctic atmospheric circulation can be more sensitive to sea ice changes.

## Methods

The polar‐optimized version of the Weather Research and Forecasting model (Polar WRF) version 4.1.1^32,33^ is used. The “control” run is a set of 10-winter (1 December to end of February next year) simulations from 2006/2007 to 2015/2016 initialized every day at 00 UTC, consisting of 903 realizations. The sensitivity simulation set, hereinafter “High sea ice concentration (SIC)” run, is identical to the “control” run but with increased SICs. For each grid point, the mean winter SIC difference between two periods (1979/80–1988/89 minus 2006/07–2015/16) based on the European Centre for Medium-Range Weather Forecasts (ECMWF) next-generation reanalysis ERA5^46^, was added if the difference is positive, and a SIC value was set so that it does not exceed 100%. Here the “sea ice increase-type” runs were used for sensitivity simulations, therefore, we can avoid any assumptions on surface temperature fields over the newly opened sea that would be needed for a “sea ice reduction-type” experiment. The responses in the atmosphere are obtained by extracting a “High SIC” simulation field from the corresponding control simulation field.

The standard model simulation is a 48 h run which is in a conventional range for a mesoscale simulation. It is long enough for allowing model spin-up of the hydrologic cycle and the boundary layer processes and short enough for the simulated climate to benefit from the initial conditions. Simulations were extended to 288 h (12 days) only when there is a strong high-pressure or low-pressure anomaly over the Barents Sea region. The high-pressure and low-pressure cases are defined as the top and bottom 5% of the 903 realizations based on 500 hPa geopotential height over 70–80°N, 15–75°E region at 24–48 h of model integration time.

Besides SIC, the “control” and “High SIC” runs use the same model configurations and background data. The simulations were run for a domain of 300 × 300 grids on a polar stereographic projection with a 24-km horizontal resolution, which encompasses the entire Arctic region north of 57°N. 70 vertical levels were defined on a terrain‐following sigma coordinate with 10 hPa top-level, corresponding to a resolution about 50 m near the surface and 440 m near the tropopause. The initial and boundary conditions for the simulations were taken from the 6 hourly atmospheric fields of the National Center for Environmental Prediction (NCEP) Final Operational Global Analysis data^47^. The sea ice albedo, sea ice thickness, and snow depth over sea ice are from the Arctic System Reanalysis version 2 in which satellite retrievals of the quantities are assimilated^48^.

## Supplementary Information

Supplementary Figures.
