# Supplementary material for: Influence of the recent winter Arctic sea ice loss in short-term simulations of a regional atmospheric model
Source: Sci Rep. 2022 May 26;12:8901. doi: 10.1038/s41598-022-12783-4 (PMC9135704; doi:10.1038/s41598-022-12783-4)
Supplement: Supplementary file 1 — Supplementary Figures. [file 41598_2022_12783_MOESM1_ESM.pdf]

SUPPLEMENTARY FIGURES FOR

Influence of the recent winter Arctic sea ice loss in short-term simulations of a regional atmospheric model

Heeje Cho[1], **Jong-Seong Kug**[2,*], **and Sang-Yoon Jun**[1,*]

[1]Division of Atmospheric Sciences, Korea Polar Research Institute, Incheon, 21990, South Korea
[2]Division of Environmental Science and Engineering, Pohang University of Science and Technology, Pohang, 37673, South Korea
[*]jskug@postech.ac.kr, syjun@kopri.re.kr

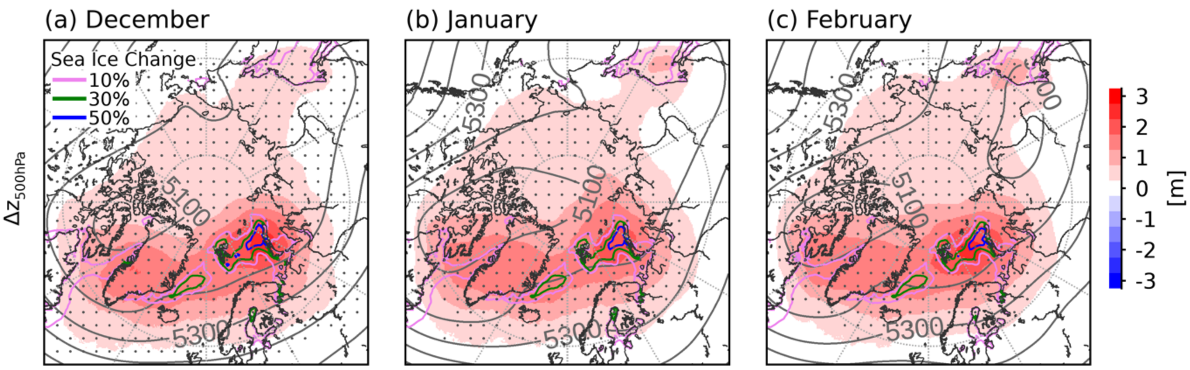

Figure S1. Responses in 500 hPa geopotential height at day 2 of model integration (24-to-48-hr) for (**a**) December, (**b**) January, and (**c**) February. Differences that are statistically significant based on a Student's *t* test at the 99% confidence level are stippled. Colored contours indicate the SIC differences between the "control" and "High SIC" runs. Gray contours are mean 500 hPa geopotential field for the control run. The map has been created using Matplotlib Basemap Toolkit ver. 1.3.0 (https://matplotlib.org/basemap/).

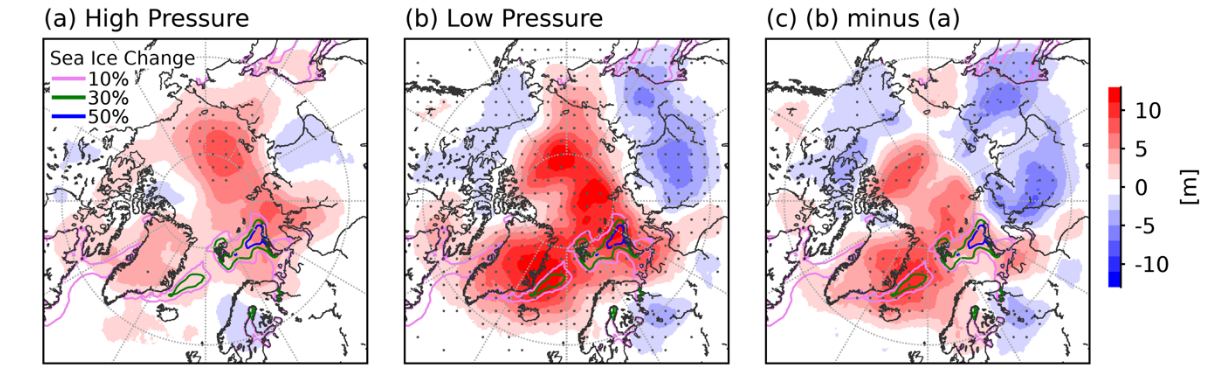

Figure S2. (**a, b**) Responses in 500 hPa geopotential height at day 7 of model integration (144-to-168-hr) for (**a**) "high-pressure" cases, and (**b**) "low-pressure" cases. (**c**) Difference between the mean response of the "high-pressure" cases and the mean response of the "low-pressure" cases. Differences that are statistically significant based on a Student's *t* test at the 99% confidence level are stippled. Colored contours indicate the SIC differences between the "control" and "High SIC" runs. The map has been created using Matplotlib Basemap Toolkit ver. 1.3.0 (https://matplotlib.org/basemap/).

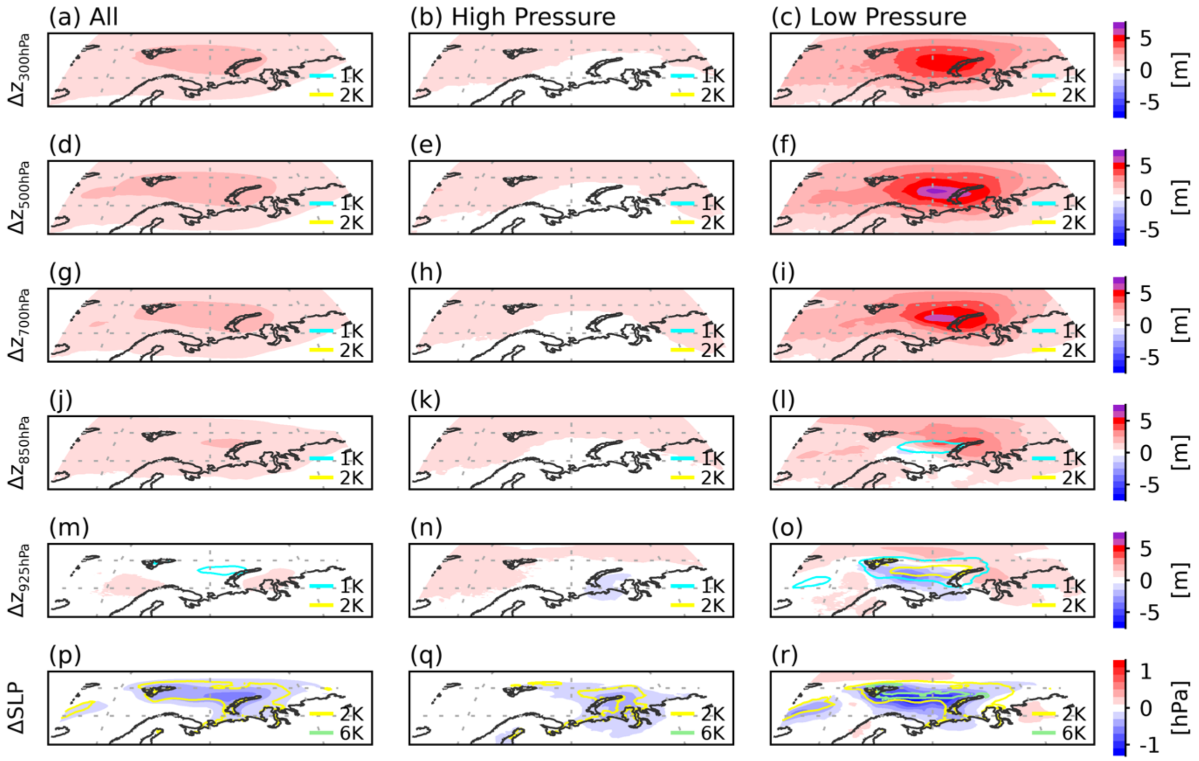

Figure S3. Responses in geopotential height (shadings in (**a**–**o**)), sea-level pressure (shadings in (**p**, **q**, **r**)), atmospheric temperature (contours in (**a**–**o**)), and surface temperature (contours in (**p**, **q**, **r**)) near the Barents Sea region; for all (**a**, **d**, **g**, **j**, **m**, **p**), "high-pressure" (**b**, **e**, **h**, **k**, **n**, **q**), and "low-pressure" (**c**, **f**, **i**, **l**, **o**, **r**) cases. Note that the contour values are different for surface (**p**, **q**, **r**) and for atmosphere (**a**–**o**). The map has been created using Matplotlib Basemap Toolkit ver. 1.3.0 (https://matplotlib.org/basemap/).

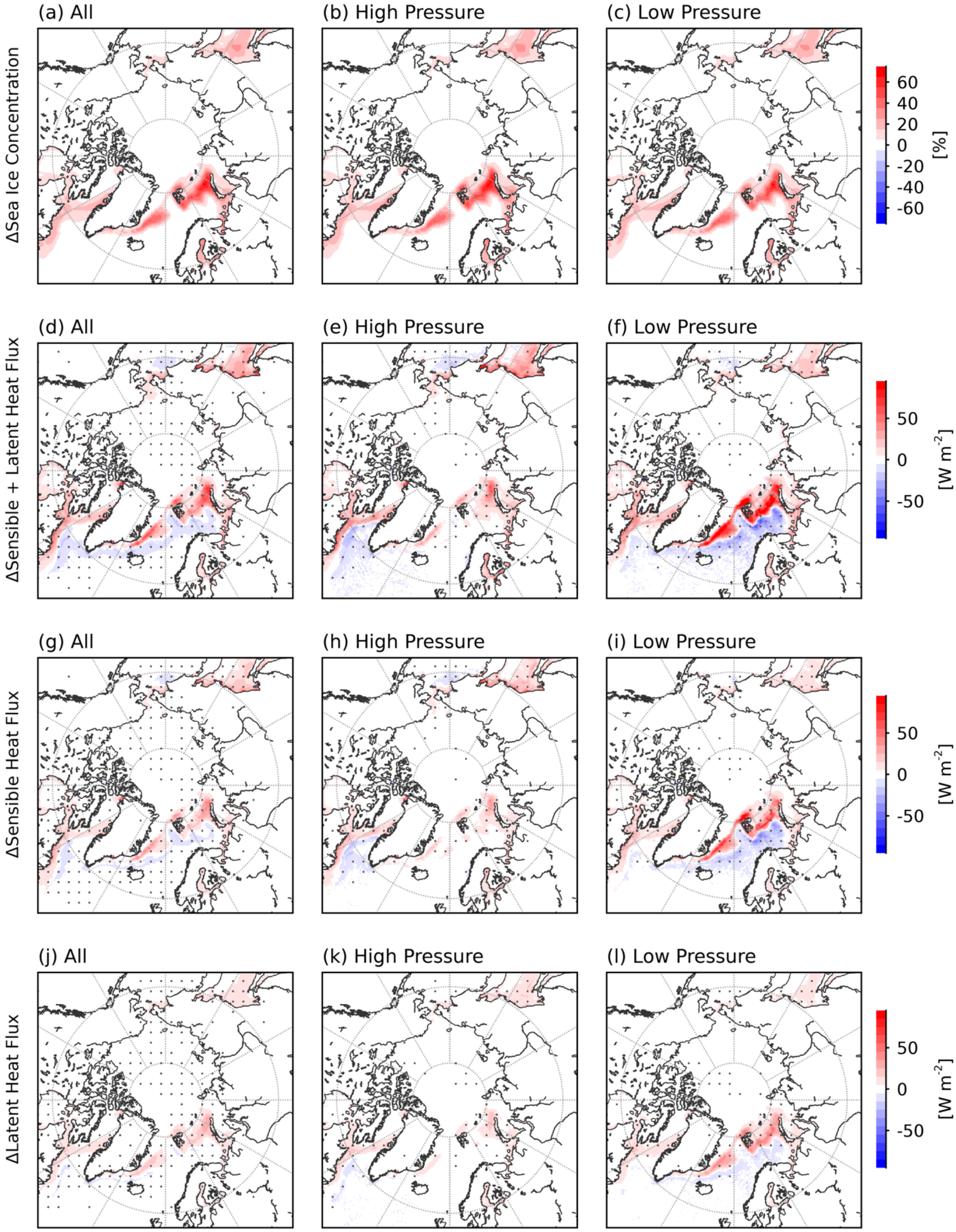

Figure S4. (**a**, **b**, **c**) Average SIC forcing applied to the "High SIC" runs. (**d–l**) Responses in total turbulent heat flux (**d**, **e**, **f**), sensible heat flux (**g**, **h**, **i**), and latent heat flux (**j**, **k**, **l**) at day 2 of model integration (24-to-48-hr). Differences that are statistically significant based on a Student's *t* test at the 99% confidence level are stippled. First column is for all 903 realizations (**a**, **d**, **g**, **j**), second column is for "high-pressure" cases (**b**, **e**, **h**, **k**), and third column is for "low-pressure" cases (**c**, **f**, **i**, **l**). The map has been created using Matplotlib Basemap Toolkit ver. 1.3.0 (https://matplotlib.org/basemap/).

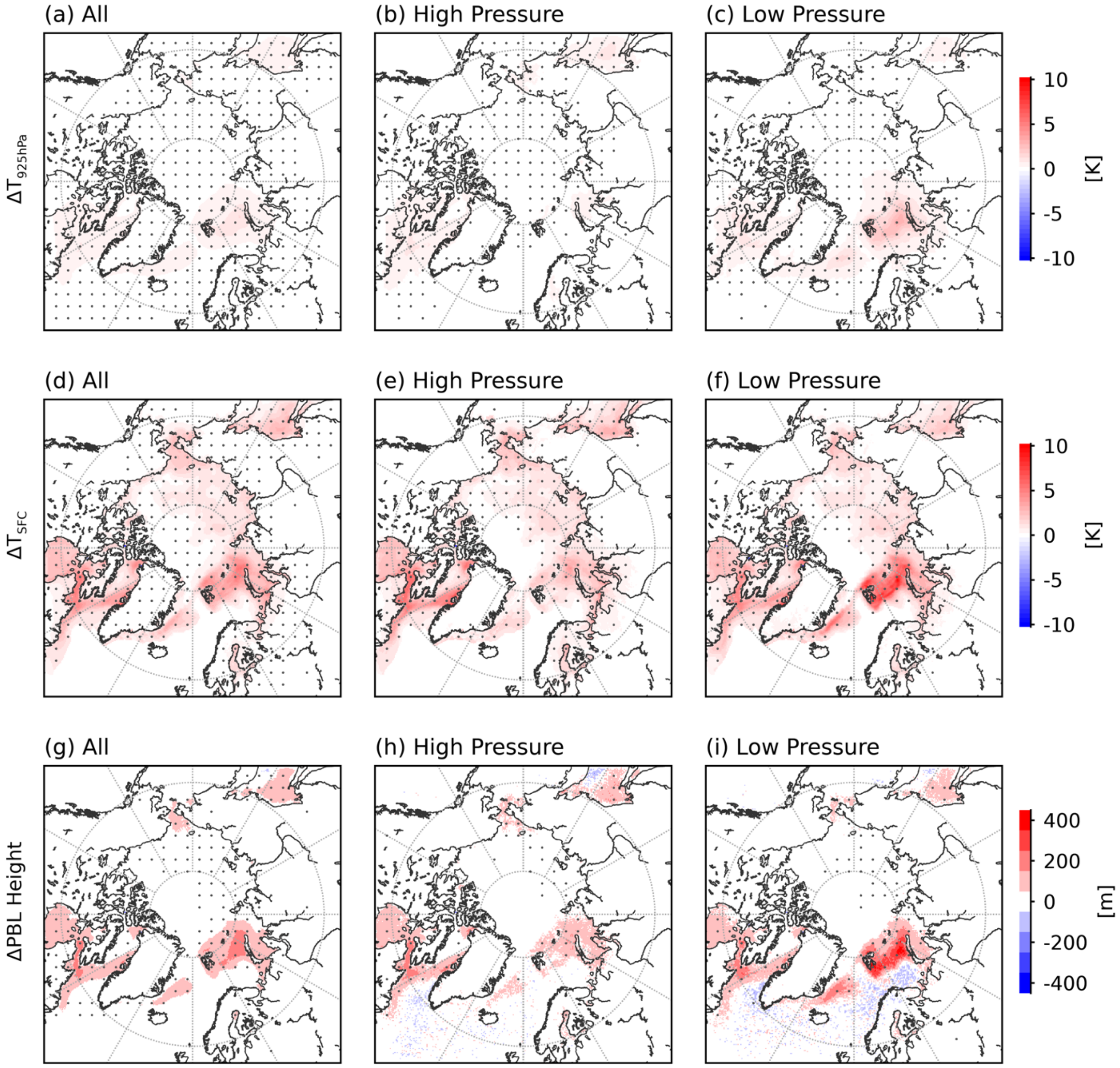

Figure S5. Responses in 925 hPa air temperature (**a**, **b**, **c**), surface temperature (**d**, **e**, **f**), and planetary boundary layer height (**g**, **h**, **i**) at day 2 of model integration (24-to-48-hr). Differences that are statistically significant based on a Student's *t* test at the 99% confidence level are stippled. First column is for all 903 realizations (**a**, **d**, **g**), second column is for "high-pressure" cases (**b**, **e**, **h**), and third column is for "low-pressure" cases (**c**, **f**, **i**). For the low-pressure cases, the PBL height increases over the sea ice loss region of the Barents Sea (**i**), but at just south of that region which is open ocean for both "control" and "High SIC" experiments, a slight decrease of PBL height appears, probably due to the near-surface atmospheric warming (**c**). This likely results in the decreases in the turbulent heat fluxes at the south of the sea ice regions (Figs. S4**d**–S4**l**). The map has been created using Matplotlib Basemap Toolkit ver. 1.3.0 (https://matplotlib.org/basemap/).
